# Promoting health and social equity through family navigation to prevention and early intervention services: a proof of concept study

**DOI:** 10.1186/s12889-022-14320-4

**Published:** 2022-10-27

**Authors:** Jeffrey Waid, Olivia Tomfohrde, Courtney Kutzler

**Affiliations:** 1 School of Social Work, University of Minnesota - Twin Cities, 1404 Gortner Ave, 105 Peters Hall, 55108 St. Paul, MN USA; 2 Family Social Science, University of Minnesota – Twin Cities, 1985 Buford Ave, 55108 St. Paul, MN USA; 3 School of Public Health, University of Minnesota – Twin Cities, 420 Delaware St SE, 55455 Minneapolis, MN USA

**Keywords:** Children, Equity, Families, Health, Inequality, Maltreatment, Navigation, Prevention, Social Work

## Abstract

**Background:**

Health and social inequality are associated with multiple adverse childhood experiences including poverty, mental illness, and child maltreatment. While effective interventions currently exist for many health and social problems, large segments of the population experience barriers accessing needed services. In alignment with broader public health efforts to reduce health and social inequality in one state in the U.S.A., the current study describes the development and formative evaluation of a brief, low cost, portable model of prevention-oriented family service navigation called *Navigate Your Way.*

**Methods:**

Caregivers of children experiencing significant unmet health or social service needs were recruited to the study. Participants completed an initial and closing telephone interview which included measures of past and current family health and social service utilization, service barriers, parenting stress, and child internalizing/externalizing behaviors. Between interviews participants created a family service plan and received 10 weeks of telephone and web-mediated family navigation, at which time process and fidelity of implementation data were collected. Frequency and descriptive statistics are provided for participant demographic characteristics, service barriers, intervention engagement, and primary and secondary study outcomes. Paired samples t-tests examined changes in study outcomes between initial and closing telephone interviews.

**Results:**

Thirty two caregivers enrolled, twenty-nine completed the study. The age range was 20–59 (M = 39.5, SD = 10.0). The majority identified as female (96.9%, n = 31), racial/ethnic minority (56.2%, n = 18), and reported an average 10 barriers to care (M = 10.4, SD = 4.1). The most frequently reported service needs were mental health care, housing, food security, transportation, and health insurance. The mean duration of intervention delivery was 83 days. Most participants (82.8%, n = 24) were connected to one or more health or social services. Caregivers reported significant improvements to youth internalizing behaviors (d = 2.5, p = .05) and high levels of overall satisfaction with the navigation approach.

**Conclusion:**

Telephone and web-mediated service navigation is a feasible and practical approach to supporting families in rapidly connecting to health and social care. Future research investigating the efficacy and implementation of *Navigate Your Way* in routine settings is indicated.

**Supplementary Information:**

The online version contains supplementary material available at 10.1186/s12889-022-14320-4.

## Introduction

A key challenge facing the public health field is ensuring communities have equitable access to health and social care. Health and social inequality disproportionately affect low income, rural, and racial and ethnic minority communities [1] and are associated with multiple adverse life course experiences including poverty, mental illness, child maltreatment, and public systems involvement [2, 3]. The consequences of health and social inequality are especially problematic for children, who are particularly vulnerable to the developmental effects of toxic stress and exposure to cumulative risk [4–6].

The ability to access health and social care is associated with improved physical, social, emotional, and behavioral health, and improved quality of life [7]. For families with children, research suggests the benefits of health and social interventions can be potentiated through delivery at key developmental periods [8]. Routine access and engagement with health and social care has been shown to promote children’s brain development, social-emotional skills, self-efficacy, and learning, as well as promote positive parenting practices and the parent-child bond [9]. Yet despite widespread knowledge of the importance of early access and engagement with health and social care many children and families experience barriers accessing needed services [10]. These barriers are often multidimensional and complex, and include individual, organizational, community, and systems-level contributors [11]. Within the United States, recognition of the need to enhance the efficacy and availability of health and social services is reflected in recent federal legislation which prioritizes the use of evidence-based practices and establishing statewide infrastructure to support the delivery of community-based services to children and families before non-voluntary interventions are indicated [12].

Recent years have seen the development of interventions designed specifically to engage and support individuals and families with identifying and connecting to health and social care. These models of case management, commonly referred to as patient navigation or service navigation, emerged with a recognition that health and social service systems are complex, fragmented, and difficult to navigate, and these difficulties can contribute to disparities in service access and underutilization of needed services [13]. Navigation approaches are diverse and have employed lay persons (e.g., peers, parents, and other service users), professionals, (e.g., community health outreach workers, case managers, social workers, nurses), and multidisciplinary teams [14]. Research with navigator models in primary care and community mental health settings suggest service navigation can improve treatment adherence, engagement with follow-up care, reduce morbidity, and improve quality of life [15].

The current evidence based for service navigation is promising, however many gaps and challenges remain. Navigator models identified in the research tend to be embedded in settings where individuals are already receiving some form of health or social care. This limits the ability to engage and support families who are disconnected from health and social care or where health care or social services exist without navigation support. Navigator models also tend to prioritize service access for individuals experiencing specialized or chronic health and mental health conditions, limiting the potential for navigation to engage and connect families early and prevent morbidity and adverse experiences altogether. Finally, while logistical barriers such as time, geography, and cost are known impediments to service access, the majority of evidence-based navigator models are delivered in-person. This can be difficult for engaging time constrained and geographically dispersed families. The additional investment of time and fiscal resources associated with in-person navigation can also be an impediment to resource constrained communities and organizations who wish to implement navigator models in their own communities.

Prevention-oriented public health approaches that reduce barriers and facilitate access to health care and social services are critically needed. Efficacious, low cost, portable, navigator models could have the potential to prevent or reduce the severity of illness, improve family well-being, and reduce the occurrence of adverse childhood experiences over time. If found effective in large scale tests, prevention-oriented navigator models may hold potential to reduce health and social inequality at the population level.

## Study purpose

The current study draws upon theoretical developments in the areas of personalized prevention [16] and experimental therapeutics [17] to evaluate a low cost, portable, manualized model of telephone and web-mediated family service navigation called *Navigate Your Way*. The study aims were threefold. First, we planned to evaluate the feasibility and acceptability of the model by recruiting and delivering navigation services to a sample of families experiencing health or social inequality. Feasibility would be determined by examining rates of recruitment, retention, and intervention engagement. Acceptability would be determined by participants’ responses to open-ended questions about their satisfaction with the navigation services they received. Our second aim was to explore the utility of the model for promoting service access and improving family well-being. Service access would be evaluated by examining the number of barriers families reported at the initial and closing assessments, and the number of connections families made to health and social care providers at the closing assessment. Family well-being would be evaluated by examining measures of parenting stress and children’s mental health at initial and closing assessments. Finally, our third aim was to identify ways to improve the model for future research. The specific research questions were: Is *Navigate Your Way* a feasible approach to engaging families and supporting early access with health and social care? Were changes to service barriers, service access, parenting stress, and children’s mental health observed among study participants? What changes are needed to enhance and improve the model?

Based on prior research of service barriers and approaches to service navigation [11, 14] we hypothesized *Navigate Your Way* would be feasible and satisfactory to study participants. Participant satisfaction would be evidenced by high rates of intervention engagement, study retention, and self-reported satisfaction with the navigation services participants received. We also hypothesized *Navigate Your Way* would promote service access and improve family well-being. Specifically, we hypothesized the informational support and resources navigators provided to the participant would assist them in locating and engaging needed services, while emotional and appraisal support provided to the primary caregiver would result in reductions to parenting stress and improvements to children’s mental health. These outcomes would be evidenced by high post-test rates of service access, and significant pre-test post-test improvements to measures of service barriers, parenting stress, and children’s mental health. Finally, as a proof-of-concept study we expected to find ways to improve the model, including enhancements to recruitment and retention, data collection, and fidelity of intervention implementation.

## Model description

*Navigate Your Way* was developed as a voluntary, telephone and web-mediated model of service navigation to engage and assist caregivers of children under 18 with accessing health care and social services for their children and family. The goals of the model were to support early access and engagement with health and social care, promote child and family well-being, and reduce the downstream occurrence of adverse childhood experiences which are commonly associated with health and social inequality. Navigation services were delivered by five research assistants who were pursuing graduate-level training in social work or marriage and family therapy. Supervision and oversight of the navigators was provided by the project’s Principal Investigator. Prior to engaging clients, navigators received pre-service training on the ethical conduct of research, protection of human subjects, project-specific research and intervention protocols, and data collection and storage procedures. Navigators also received weekly team supervision on the theoretical orientations guiding implementation of the model, and specific activities and strategies to engage and support caregivers during the navigation process. Specific navigation activities included a comprehensive assessment of the family system, their service needs, and service barriers; co-creating a plan with the primary caregiver to identify, prioritize, and access personalized health and social care for their children and family; weekly check-ins with the caregiver to support implementation of their service navigation plan; and a closing assessment and discharge plan. The total duration of a caregiver’s participation in the study was 12 weeks. Major assessments were conducted by telephone during week 1 and week 12, with check-ins conducted weekly by telephone, text message, and email between weeks 2–11. All contacts with the participant were scheduled at days and times that were convenient to the caregiver. This flexible, person-centered approach was designed to reduce logistical barriers, foster client engagement, contain project costs, and widen programmatic reach.

The *Navigate Your Way* model draws upon theories of self-determination [18], empowerment and advocacy [19], social justice [20], anti-oppressive practice [21], and person-centered care [22]. Navigators engaged each participant using a relationally oriented [23] and strengths-based perspective [24]. Interactions with the participant were solution-focused [25] and task-centered [26]. Navigators provided support to caregivers through a series of activities which included comprehensive and ongoing assessment of the family’s service needs and barriers, collaborative goal planning, service research, community outreach, parent education, progress monitoring, collaborative problem solving, and social support. These programmatic elements have previously been identified in a variety of promising and efficacious models of service navigation [11, 14].

## Research methods

The proof of concept study was developed as a single-group mixed-methods research design. To determine model feasibility and establish preliminary estimates for continuous outcome measures, thirty caregivers were targeted for recruitment. This sample size is typically considered sufficient for studies examining the feasibility of health care services interventions [27] and consistent with formative evaluations of other service navigation programs [13]. Individuals were eligible for inclusion if they were the primary caregiver of one or more children under age 18, experiencing significant or persistent barriers to health and social care for their children and family, were not currently receiving case management services, were not actively involved with the child protection or juvenile justice system, had a telephone to make and receive phone calls, and planned to reside in the state where the research was conducted for the duration of their participation.

Participants were recruited to the research through community partnerships and direct to consumer advertising. Community recruitment partners included three organizations; an online high school serving families from 15 school districts, one rural county department of human services, and one urban Head Start program. Direct to consumer advertising included one statewide 12-day public radio advertising campaign and a continuous targeted social media marketing campaign. In all instances individuals who were notified of the study self-referred to the research and completed a brief telephone screening interview to determine their eligibility for enrollment. Written informed consent was obtained from eligible individuals electronically via an online survey platform. Participants received $25 for each telephone interview and $5 for each check-in, and could receive up to $100 for their participation in the study. Institutional Review Board approval and oversight was provided by the University of Minnesota – Twin Cities, study #7787.

### Participant flow and demographic characteristics

**Fig. 1:**
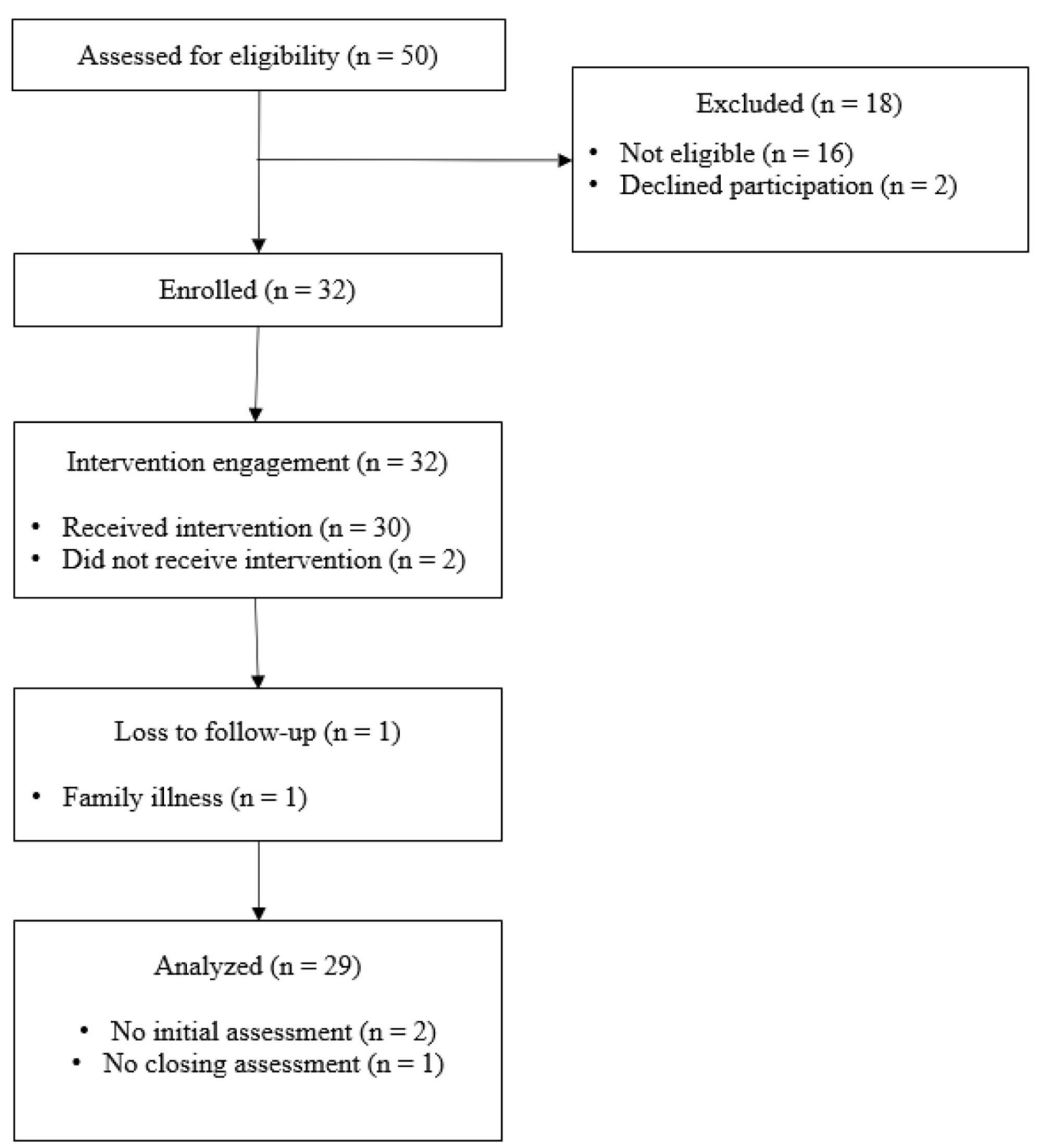
Flow diagram of participant enrollment and retention

The study received 109 inquiries, conducted 50 screening interviews, and enrolled 32 participants. All of the participants were caregivers of children under age 18 who had an unmet health care or social service need, were experiencing significant or persistent barriers accessing needed services, and were residing in the state where the research was conducted. The age range was 20 to 59 for participating caregivers and infant to 17 for the target child. The sample was racially and ethnically diverse, with more than half of caregivers and nearly three-quarters of children identifying as racial or ethnic minority. All but one caregiver identified as female. Just under two-thirds of target children were also female. Table 1 summarizes participant demographic characteristics (Additional file 1a & 1b). Figure 1 describes the participant flow and retention (Additional file 2).

**Table 1:** Summary of demographic characteristics for the full sample and by retention status

Demographic Characteristics	Full sample (n = 32)		Study completers (n = 29)		Lost to attrition (n = 3)		
	M	SD	M	SD	M	SD	
Caregiver age	39.5	10.0	38.6	9.7	49.0	8.2	
Child age	10.1	5.8	10.1	5.9	10.3	4.9	
Children in home	1.9	1.0	1.9	1.0	1.7	0.58	
Household size	3.5	1.3	3.5	1.4	4.0	1.0	
Caregiver race	%	N	%	N	%	N	
White	43.8	14	44.8	13	33.3	1	
African-American	40.6	13	41.4	12	33.3	1	
Latino	3.1	1	0.0	0	33.3	1	
Asian / Pacific Islander	3.1	1	3.4	1	0.0	0	
Multiracial	6.3	2	6.9	2	0.0	0	
East African	3.1	1	3.4	1	0.0	0	
Caregiver Gender							
Female	96.9	31	96.6	28	100.0	3	
Male	3.1	1	3.4	1	0	0	
Child Race							
White	28.1	9	31.0	9	0	0	
Black	37.5	12	37.9	11	33.3	1	
Latino	6.3	2	3.4	1	33.3	1	
Asian/Pacific Islander	3.1	1	3.4	1	0	0	
Multiracial	21.9	7	20.7	6	33.3	1	
Child Gender							
Female	62.5	20	65.5	19	33.3	1	
Male	37.5	12	34.5	10	66.7	2	

*Note.* Child race data were missing for one participant. No significant differences were noted for demographic characteristics across study completers and participants who were lost to attrition

### Measures

#### Participant intake form

The *Participant Intake Form* was a 90-item measure designed to facilitate a comprehensive assessment of the family unit. Domains included participant (caregiver) and target child demographic information, household composition, quality of household member relationships to the caregiver and target child, important non-resident individuals, quality of important non-resident individuals’ relationships to the caregiver and target child, presenting problems, current and past service utilization, service needs/barriers for the child and family members across major health domains (e.g., mental health, medical, dental, vision, speech and hearing), educational and vocational service needs, basic needs (e.g., housing stability, food security, clothing), family community involvement (e.g., non-health or social service community engagement), and cultural considerations (e.g., religious, immigration, languages spoken, cultural service preferences). The participant intake form was completed once during the study at the beginning of the initial telephone interview.

#### Service barriers checklist

The service barriers checklist was a study-specific, 23-item measure designed to assess the breadth and severity of service barriers across five domains: family factors (e.g., lack of clarity about service needs, ambivalence/lack of motivation to engage services, feelings of shame/stigma, family attitudes/discrepancies about what to do), logistical factors (e.g., insurance/financial issues, time, work schedules, transportation barriers), provider factors (e.g., referral procedures, scheduling, availability, provider competency, specialist availability, confidentiality concerns), system factors (e.g., lack of services in geographic area, lack of cultural sensitive services) and “other” factors. Navigators completed the checklist at the conclusion of the initial telephone interview and rated the severity of identified barriers a 4-point scale (e.g., not present, minor, moderate, severe). The service barriers checklist was then used to assist the navigator and participant in identifying areas that may be important to service planning and subsequent navigation activities. At the closing telephone interview navigators completed the service barriers checklist a second time to track changes or persistence of service barriers. The number of barriers could range from 1 to 23 for total barriers, 0–9 for family barriers, 0–6 for logistical barriers, 0–6 for provider barriers, and 0–2 for system barriers. Cronbach’s alpha indicated good internal consistency for the total (pre = 0.80; post = 0.87), family (pre = 0.74; post = 0.80), and provider (pre = 0.72; post = 0.74) domains, and acceptable to weak internal consistency for the system (pre = 0.60; post = 0.24), and logistical domains (pre = 0.42; post = 0.47).

#### Family navigation service plan

The family navigation service plan was an open-ended, project-specific form that was mutually constructed by the participant and navigator at the conclusion of the initial telephone interview. The form identified and prioritized the family’s presenting concerns, service needs, and barriers to service access. The plan contained service access goals for three unmet service needs and three behaviorally focused objectives for each service access goal. Goals were added to the plan only if the need was currently unmet and the family was experiencing barriers accessing those services. The navigator assisted the client in clarifying the goals and objectives of the plan using the S.M.A.R.T. treatment planning framework [28]. The plan was finalized within one week of the initial telephone interview and delivered to the caregiver for feedback and approval. The plan served as the guiding document for subsequent navigation activities with the study participant. As participants accessed the desired services in their plan the navigator would update the plan to reflect the service plan goal was accomplished. Service access was defined as receiving the desired health care or social services that were written into their service plan. At baseline, each participant had accessed zero services in their service plan. The range of goals a participant could complete during study participation was 0–3.

#### Strengths and difficulties questionnaire (SDQ)

The SDQ is a brief 25-item measure assessing the psychological attributes of the target child across five domains. These include emotional symptoms, conduct problems, hyperactivity and inattention, peer relationship problems, and prosocial behavior [29]. A three-subscale division for internalizing problems (emotional and peer problems), externalizing problems (conduct and hyperactivity/inattention problems) and prosocial behavior has been validated and recommended for use with the general population [30]. The measure also includes a five-item impact supplement assessing the frequency, severity, social impairment, and burden of problematic attributes on child and family functioning. The current study utilizes the internalizing, externalizing, and total scores for the measure at initial and closing telephone interviews. Cronbach’s alpha suggested acceptable internal consistency for the measure [SDQ total: (pre = 0.60, post = 0.76), internalizing: (pre = 0.55, post = 0.75), externalizing: (pre = 0.61, post = 0.62)].

#### Parenting stress index-4-short form (PSI-4-SF)

The PSI-4-SF is a 36-item measure designed to assess three components of the parent-child system and interactions between them [31]. The measure provides a global rating of total stress and ratings for three subscales: parental distress, parent-child dysfunctional interactions, and difficult child. High ratings for parental distress suggest adjustment difficulties which may indicate a need for caregiver-focused services. The parent-child dysfunctional interaction subscale assesses strength of the parent-child bond, potential need for family-focused intervention, and risk of future child abuse. The difficult child subscale examines temperament and behavioral indicators which might indicate adjustment difficulties and psychopathology. High ratings across subscales may indicate the need for parenting consultation or parenting classes to improve child behavior. Cronbach’s alpha suggested good internal consistency for the measure and subscales [PSI-SF total: (pre = 0.93, post = 0.91), parental distress (pre = 0.88, post = 0.83), parent-child dysfunctional interaction (pre = 0.78, post = 0.80), difficult child (pre = 0.88, post = 0.83)].

#### Navigation check-in form

The navigation check-in form was a structured case note that was completed by the navigator at the conclusion of each participant contact. The form provides a record of family navigation service plan progress and details the day, time, week of intervention, type of contact (e.g., phone, text, email), person initiating contact (e.g., participant, navigator), purpose of the check-in, related family navigation service plan objectives, check-in disposition, and navigator notes. The check-in forms encompassed all attempted and achieved contacts with the participant. and were completed once per week for the 10 weeks of navigation service delivery. For the current study a count summarizing the total number of check-ins a participant completed was recorded. The number of check-ins a participant could complete ranged from 0 to 10.

#### Closing interview open-ended questions

At the conclusion of the study participants were asked to provide feedback about their experience and satisfaction with navigation services they received through a closing telephone interview. Open-ended questions included: what is your impression of the family navigation service? Were certain elements that were particularly helpful? Were there particular elements that were not helpful? What would you recommend be changed to better improve the service? Would you recommend this service to others? Why or why not?

#### Navigator effort

Throughout the course of the study each navigator maintained a spreadsheet that recorded all project related activities they engaged in. The form detailed the date of the activity, total time devoted to the activity (in minutes), and a description of the activity. Activities were project-wide (i.e., training, project development, administrative activities, supervision), and client-specific (i.e., recruitment, screening, and enrollment activities, encounter preparation, client outreach, service research, initial telephone interview, check-in’s, closing telephone interview).

#### Navigation discharge reports

After the conclusion of the closing telephone interview navigators were asked to complete a brief report summarizing their work with each study participant. The report was designed to provide navigators with a space to capture their reflections, insights, and lessons learned in their work with and on behalf of the participant. Navigators were asked to consider the following questions as they drafted their reports: What navigation activities did you engage in with the participant directly? What navigation activities did you engage in on behalf of the participant? What other project activities did you engage in? What navigation activities do you think are critical to helping families connect to services and should be emphasized in training/case consultation? What were the most difficult challenges families presented with that were most difficult to address? What elements of the protocol didn’t seem to work very well that should be revised or removed?

### Analytic Strategy

To answer the research questions a concurrent mixed methods approach to data analysis was undertaken. Quantitative analysis included frequency and descriptive statistics for participant characteristics, and paired samples t-tests for service barriers, service access, parenting stress, and children’s mental health. Qualitative data were analyzed using a hybrid inductive and deductive coding approach [32] Quantitative and qualitative results were then mixed during data interpretation [33].

To assess the feasibility and acceptability of the model, frequency and descriptive statistics were calculated for rates of participant retention and attrition, participant engagement with navigation check-ins, navigator adherence to the 12-week study timeline, and navigator effort across project-wide, client-specific, and total project activities. To assess participants satisfaction with the model a hybrid thematic analysis of participant’s open-ended responses to the questions “What is your impression of the family navigation service?” and “Would you recommend this service to others? Why or why not?”

To examine rates of service access for the sample cross-tabulations were calculated for service access by service type. Paired samples t-tests were then calculated to determine if any observed changes to service access, parenting stress, and children’s mental health were statistically significant. Paired samples t-tests were conducted for service plan goal completion, service barrier checklist total and subscale scores, PSI-SF total and subscale scores, and SDQ total and subscale scores.

To determine what changes were needed to improve the model, a hybrid inductive and deductive thematic analysis of participant responses to the question “What would you recommend be changed to better improve the service?” and navigator responses to the discharge note question “what elements of the protocol didn’t seem to work very well that should be revised or removed?” were conducted.

## Results

### Feasibility, acceptability, and participant satisfaction

The project successfully retained 90.6% (n = 29) of study participants. Two did not complete the initial telephone interview and were not responsive to navigator outreach attempts, another withdrew during the course of intervention due to reasons unrelated to the study. Participant engagement with the intervention was otherwise high, with study completers participating in an average of 9 (M = 9.0, SD = 1.4, Range = 6–10) check-ins. Navigator adherence to the 12-week project timeline was good. The mean duration of intervention delivery was 83 days (M = 82.7, SD = 9.0, range = 74–105), with the majority of participants (68.9%, n = 20) completing the intervention within 7-days of their planned study termination date.

Navigators recorded 904 data points related to their effort on the project. The most frequently reported client-specific activities were participant check-ins, service research, and client outreach. The most frequently reported project-wide activities were supervision, administrative activities (e.g., data entry), and recruitment & enrollment. The distribution of navigator effort and average time devoted to each project activity are detailed in Table 2 (Additional file 3a & 3b).

**Table 2:** Summary of navigator effort and time devoted to each project activity

Activity	Effort Distribution		Average Time Per Activity			
	%	N	M	SD	Min	Max
Check-in	25.0	226	24.3	15.0	2	105
Service research	15.0	136	35.3	21.9	10	120
Client outreach	17.9	162	18.4	19.2	2	180
Supervision	10.1	91	92.3	33.1	10	150
Administrative activities	9.4	85	35.6	27.9	5	120
Recruitment & enrollment	6.5	59	54.3	78.4	1	420
Pre-service training	3.3	30	68.5	45.2	30	190
Initial interview	3.2	29	62.8	30.0	5	120
Closing interview	2.8	25	50.0	23.2	20	120
Client preparation	2.5	23	45.2	33.5	10	150
Discharge activities	2.2	20	44.0	19.4	15	65
Family service planning	2.0	18	38.6	30.7	10	130

*Note*: Data were missing for family service planning (n = 12), closing interview (n = 4), and initial interview (n = 1)

In closing telephone interviews all 29 study completers reported they were satisfied with the model and would recommend *Navigate Your* Way to other caregivers in need of assistance accessing health care and social services for their children and family. When asked why, emergent themes included that the program was supportive in connecting caregivers to needed resources, and connection to resources positively impacted the well-being of the caregiver, the target child, and other household members. Participants reported: “I got resources I needed and fast. I didn’t have to wait 6 months” (Participant 111). “I appreciated the opportunity to work every week and have an ally to support and encourage me on a weekly basis” (Participant 122). “It was helpful for a lot of things I couldn’t focus on with just myself” (Participant 117). ““The way the pandemic has been, there are a lot of single parents like myself, and even when the person (navigator) doesn’t know you personally, they can help support that individual in helping find the resources they need “ (Participant 113). Participants also noted the benefits of having weekly support for accessing needed care: “The navigator would call every week so it kind of kicked me in gear, the weekly call once a week–I think that’s what really helped me. This was something I had been needing to do for 2 years and actually was able to do in 12 weeks and get it done!“ (Participant 121). “I couldn’t find the time to get the resources to make the calls so with you giving them to me I was able to have them in my email and go back to them. I like that you kept persistently calling even when I didn’t answer. (Participant 115).

Regarding benefits to the caregiver and family, participants remarked: “I learned a lot (about how to discover and follow-up on resources)” (Participant 130). “You called and we got the paperwork done. We got (target child) in and she was the one we were most concerned about” (Participant 125). “You searched for resources and provided these for me, and I was able to follow up successfully. My (household member) benefitted from this by gaining employment, and I did as well” (Participant 126). “I know that being a parent, specifically a single parent, is challenging in and of itself, and sometimes we can need things but not really know how to access those particular resources. I know other people could benefit from a program; I would tell them that there is a program that could help connect them to resources if they need it; it’s a little more personal than just getting connected. Is the personal touch makes the difference” (Participant 104). “I thought it was really great that there was somebody there to help me find things faster than if I were to try these things on my own. It was nice to hear a human voice to talk through these things with someone (Participant 103).” Data supporting these conclusions are available in Additional file 4.

### Primary and secondary participant outcomes: quantitative results

Primary and secondary study outcome measures and paired samples t-test results are reported in Table 3 (Additional files 1a & 1b). Participants each reported three service needs in their family navigation service plan. Two participants withdrew before the service plan could be completed, which provided a total of 90 service needs at the baseline assessment. Descriptively, participants reported service needs were mental health (28.9%, n = 26), housing (13.3%, n = 12), food security (10%, n = 9), transportation (8.9%, n = 8), health insurance (8.9%, n = 8), healthcare (6.7%, n = 6), child care (4.4% n = 4), vocational training/employment (3.3%, n = 3), school-based services (3.3% n = 3), diapers (3.3% n = 3), child social skills (3.3% n = 3), financial support (2.2%, n = 2), parenting education (2.2%, n = 2), and winter clothing (1.1%, n = 1).

One participant withdrew before study completion, which resulted in missing service access data for mental health (n = 1), housing (n = 1), and clothing (n = 1) related service needs. Within the sample of study completers, missing data were present for one diaper (n = 1) related service need. This provided 86 records of service access at the follow-up assessment. Among study completers, participants engaged with an average of 2 out of 3 possible services in their family navigation service plan. The most frequently accessed services were mental health care, (60%, n = 15), food security (77.8%, n = 7), housing (63.6%, n = 7), health care (100%, n = 6), health insurance (75%, n = 6), and vocational training/employment services (66.7%, n = 2). Less frequently accessed services included transportation (50%, n = 4), school-based services (33.3%, n = 1), diapers (33.3%, n = 1). No study completers were able to access childcare, financial support, or child social skills programs during the study period.

Paired samples t-test results indicate the change in number of services participants accessed between initial and closing telephone interviews was statistically significant [M = 1.8, SD = 1.1, t[28] = 8.7, p = .00, d = 1.1]. Statistically significant reductions in total service barriers (M = -2.9, SD = 4.4, t[28] = -3.6, p = .01, d = − 0.66), family barriers (M = -1.4, SD = 2.5, t[28] = 8.6, p = .00, d = − 0.55), and provider barriers (M = − 0.76, SD = 1.7, t[28] = -2.37, p = .03, d = − 0.44) were observed. Statistically non-significant reductions to logistical barriers were also observed.

At baseline participants responded in the 65th percentile for parental distress (M = 29.8, SD = 11.4), 85th percentile for parent-child dysfunction (M = 34.0, SD = 7.8), and 66th percentile for difficult child (M = 30.1, SD = 10.9). Paired samples t-test uncovered no significant differences in reports of total or subscale scores, however slight increases for each measure were observed. For children’s mental health, participants scored in the 93rd percentile for total difficulties (M = 17.6, SD = 4.5). Statistically significant improvements were observed for youth internalizing (M = -1.0, SD = 2.5, t[26] = 2.1, p = .05. d = 0.41) difficulties. Statistically non-significant reductions to total difficulties were also observed.

**Table 3:** Summary of study measures and paired samples t-test test results

Outcome measures	Initial Interview		Closing Interview					
	M	SD	M	SD	t	df	p	d
Service Engagement	0.0	0.0	1.8	1.1	8.6	28	0.00	1.1
Service Barriers Checklist								
Family Barriers	4.6	2.2	3.2	2.6	-2.96	28	0.01	− 0.55
Logistical Barriers	3.0	1.4	2.5	1.5	-1.81	28	0.08	− 0.34
Provider Barriers	2.0	1.7	1.2	1.6	-2.37	28	0.03	− 0.44
System Barriers	0.76	0.78	0.55	0.63	-1.29	28	0.21	− 0.24
Total Barriers	10.4	4.1	7.5	5.2	-3.56	28	0.01	− 0.66
Parenting Stress Index - Short Form								
Parental Distress	27.3	11.4	29.8	11.4	1.69	24	0.11	0.34
Parent-Child Dysfunction	32.5	7.9	34.0	7.8	1.35	25	0.20	0.26
Difficult Child	30.1	10.9	30.1	8.4	− 0.02	26	0.98	− 0.00
Total	88.4	26.2	94.2	19.4	1.51	20	0.15	0.33
Strengths & Difficulties Questionnaire								
Internalizing	8.9	3.1	7.9	3.2	2.11	26	0.05	− 0.41
Externalizing	8.6	2.5	8.2	2.6	1.19	24	0.25	− 0.24
Total	17.6	4.5	16.2	5.3	1.91	24	0.07	− 0.38

*Note*: Missing data were present for PSI-SF total (n = 9), parental distress (n = 5), parent-child dysfunction (n = 4), and difficult child (n = 3) subscales. Data were also missing for the SDQ total (n = 5), internalizing (n = 3), and externalizing (n = 5) subscales

### Programmatic changes to enhance rigor and ecological relevance

Three participants recommended changes to the model. One participant reported some confusion initially about the aims of the research, and suggested it would be beneficial to provide additional information about what to expect in the initial screening interview. A second participant felt the standardized measures were not relevant to their situation. A third participant felt a more frequent check-in schedule than once per week could have been helpful to them.

Similar themes emerged from the analysis of navigator discharge reports. With respect to intervention intensity, navigators noted some participants were highly motivated and made steady progress each week while others could have benefited from more frequent contacts to provide support and motivation to accomplish their service goals. Navigators also recommended changes to data collection during the initial and closing telephone interviews, noting the administration of standardized assessments over the telephone detracted from the rapport they were working to establish with the caregiver. To address this, navigators recommended administering some measures online in advance of the first meeting to provide more time in the initial encounter to establish rapport and build relationships with participants. Data supporting these conclusions are available in Additional files 4 & 5.

## Discussion

Our study first hypothesized *Navigate Your Way* would be feasible and acceptable to study participants. This hypothesis was supported by very high rates of participant retention, engagement with the intervention, and unanimous recommendations among study completers to continue and expand the service to other caregivers. A recurring theme which was reflected in the closing interviews were caregivers’ appreciation for having someone available to assist them in both the relational (e.g., support, encouragement) and technical aspects (e.g., identifying, prioritizing, and planning for services) of service navigation. This was further reflected in the distribution of navigator effort, which overwhelmingly focused on activities with or on behalf of the participant.

Our second hypothesis was that participant engagement with *Navigate Your Way* would result in high rates of service access, reductions in caregiver parenting stress, and improvements to children’s mental health. These findings were partially supported. Twenty-six participants were connected to at least one service in their family navigation service plan, with the majority reporting connection to two or more services during the intervention period. Participants were connected to mental health, health care, housing, food security, health insurance, and vocational employment services at high rates. This was expected given the navigator models focus on access to health care and social services. The frequencies with which these service needs were reported also provided multiple opportunities for service access, and indicate support for the model’s focus on access to basic needs alongside more traditional forms of health and mental health care. The model also demonstrated some success connecting caregivers to transportation, school-based services, and clothing. Access to transportation was due in part to family resources and geographic availability, whereas access to clothing and school-based resources were partially dependent upon supply and organizational responses to participants’ requests. The model did not facilitate access to childcare, financial support, and social skills programs. This could be due to the particular challenges the caregiver and family were experiencing, increased demand due to the COVID-19 pandemic, low supply of available services within a community, or timelines associated with service access.

Health care and social service access may also be a result of changes to the specific service barriers navigators identified in their work with participants. For example, it is possible the reductions to family barriers were a result of the models focus on primary caregivers and their ability to effect change within themselves and in their family systems around things like prioritizing a need, building motivation, and engaging in change behaviors. Reductions to provider barriers could be due in part to navigator and participants’ mutual efforts to identify and secure providers that were a better match and had the needed expertise for the families’ specific problems. Similarly, changes to logistical barriers may be due to resource generation and collaborative problem solving efforts undertaken by the participants and their navigator. Lack of change to system level service barriers may be due to low response rates for construct items, under identification of systemic barriers, or limitations to the navigator models scope of influence on systemic barriers to service access.

Changes to parenting stress and children’s mental health were also observed. Participants reported moderate rates of parenting stress at the initial assessment, and non-significant increases to parental distress and parent-child dysfunction were observed at the closing assessment. This could be due to the timing of the study which coincided with the onset and early stages of the COVID-19 pandemic, when participants and children were often home together 24 h a day 7 days a week. It is also possible that at the initial assessment participants were more guarded in their responses to questions about parenting and parent-child conflict than they were at the closing assessment, once trust and familiarity had been established between the participant and navigator. For children’s mental health, participants reported very high rates of child mental health challenges at initial assessment, which then decreased over the course of navigation. This suggests changes to either parents’ appraisal of their child or observed improvements to their child’s mental health functioning, and indicates navigation itself may promote functional improvements in the family system. This would be consistent with the satisfaction participants reported with the navigator and the services they received and with previous studies demonstrating the potential for brief, non-clinical preventive interventions to promote mental health and well-being [34]. Taken together, Study results are promising and provide support for continued investigation into telephone and web-mediated service navigation as a public health strategy to promote service access and improve parent, child, and family well-being.

Our third hypothesis was that as a proof-of-concept study we would expect to find ways to improve the model, particularly with respect to recruitment and retention, data collection, and fidelity of intervention implementation. This hypothesis was supported. The study recruited participants using diverse strategies. Social media advertising was most successful in terms of generating interest and enrollment, suggesting direct to consumer advertising may be most effective for engaging and recruiting families experiencing health and social inequality. With respect to retention, participant feedback and analysis of the timing of participant withdrawal suggest a need to improve the clarity and focus of messaging about the study, and should be reflected in the studies marketing, recruitment, and information sharing procedures. This may be helpful to improve awareness of the service and the potential benefits of early access and engagement with family oriented health care and social services in the future.

Findings also provide insights into data collection and fidelity monitoring. Emergent themes from participant feedback and navigator discharge notes noted the tension between structured data collection and establishing and maintaining rapport in the working relationship. Bifurcating data collection into synchronous (e.g., navigator and client together) and asynchronous (e.g., client or navigator independently) components that align more intentionally with the model activities and intended outcomes could be helpful, however alternative methods of data collection would need to be established for individuals without reliable access to a computer or internet connection. Study-specific measures may also benefit from further refinement. The participant intake form was lengthy and could be reduced to focus more narrowly on questions specific to prior and current service utilization. The service barriers checklist could benefit from additional psychometric testing and refinement, particularly with regard to items assessing logistical and system level service barriers. With respect to fidelity monitoring, navigator effort and discharge reports provide insights into areas where more focused measurement of important operating mechanisms (e.g., rapport, support, motivation, problem solving, self-efficacy) and their relationship to key intervention activities (e.g., interviews, check-ins, service research) is warranted.

## Limitations

A number of limitations should be noted. The first limitation is the current study employed a single-group design, so all participants were allocated to receive the intervention and were not compared to a control condition or services as usual. To address this future research should evaluate the comparative efficacy of *Navigate Your Way* to a comparison condition. Navigators and participants both reported increased frequency of check-ins could be beneficial for some participants and circumstances, so it may be ideal to test the efficacy of the model using an adaptive intervention design [35]. Relatedly, the proof-of-concept study enrolled only 32 caregivers, and data were collected from only two reporting sources (i.e., participants, navigators) over a 12 week period. This can be addressed by recruiting a larger sample, taking measurements from additional reporting sources (e.g., supervisory or peer review of a selection of participant contacts or exit interviews) over an extended period of time (e.g., 6-month and 12-month follow-along). Expanding the types of data collected (e.g., child maltreatment reports, inpatient hospitalizations, arrests) and key operating mechanisms relevant to service access and family well-being can further strengthen empirical support and ecological relevance of the model, and provide additional support for telephone and web-mediated family service navigation and public health promotion and prevention strategy. Finally, participants received incentives at initial and closing interviews and through weekly navigator check-ins. This could inadvertently bias engagement with intervention activities and potentially influence project outcomes. To address this future studies should provide incentives at major assessment intervals and remove incentives from activities connected to the intervention itself. This would reduce the potential for incentive payments to influence intervention engagement and service access outcomes. Including additional closing interview questions asking participants about their willingness to engage without research incentives would also provide additional insights into the long-term sustainability of the model.

## Conclusion

The current study provides preliminary support for the feasibility and acceptability of *Navigate Your Way* as a public health approach to support early family access and engagement with health care and social services. Results also provide support for service navigation as an approach to support parent and child well-being, which can be achieved through connection to traditional health care services and other forms of support, including non-clinical services and those designed to meet basic needs. Future research should test the comparative efficacy of *Navigate Your Way* to services as usual on service access and child and family well-being. This research should include a parallel process of community stakeholder engagement and a cost-benefit analysis of navigation practices. A pilot study of this nature would provide important information about key mechanisms within the model that drive access to care, generate information to support legislative and policy actions to reduce structural barriers to care, and promote sustainability of efficacious practices derived from the model in routine settings.

## Electronic supplementary material

Below is the link to the electronic supplementary material.

Supplementary Material 1

Supplementary Material 2

Supplementary Material 3

Supplementary Material 4

Supplementary Material 5

Supplementary Material 6

Supplementary Material 7

## Data Availability

All data generated or analyzed during this study are included in this published article as supplemental information files. Additional File1a.xlsx and Additional File1b.pdf include participant characteristics and primary and secondary study outcomes. Additional File 2.xlsx include participant recruitment and selection. Additional File 3a.xlsx and Additional File 3b.pdf include navigator effort. Additional File 4.xls include closing interview open-ended questions. Additional File 5.xlsx include navigator discharge notes.
