# Supplementary material for: Promoting health and social equity through family navigation to prevention and early intervention services: a proof of concept study
Source: BMC Public Health. 2022 Oct 27;22:1972. doi: 10.1186/s12889-022-14320-4 (PMC9610316; doi:10.1186/s12889-022-14320-4)
Supplement: Supplementary file 2 — Supplementary Material 2 [file 12889_2022_14320_MOESM2_ESM.pdf]

*Promoting health and social equity through family navigation to prevention and early intervention services: A proof of concept study
*Principal Investigator: Jeffrey Waid
*Contact email: jdwaid@umn.edu

```
*******************************************************
*PARTICIPANT, CHILD, AND HOUSEHOLD CHARACTERISTICS
*******************************************************

*Caregiver age.

DESCRIPTIVES VARIABLES=intake2
/STATISTICS=MEAN STDDEV MIN MAX

*Target Child Age

DESCRIPTIVES VARIABLES=intake6
/STATISTICS=MEAN STDDEV MIN MAX.

*Number of Children in Home Including Target Child.

DESCRIPTIVES VARIABLES=ChildrenInHome
/STATISTICS=MEAN STDDEV MIN MAX.

*Household Size Including Participant and Target Child.

DESCRIPTIVES VARIABLES=HouseholdToatal
/STATISTICS=MEAN STDDEV MIN MAX.

*Caregiver Race.

FREQUENCIES VARIABLES=ParticipantRace
/ORDER=ANALYSIS.

*Caregiver Gender.

FREQUENCIES VARIABLES=ParticipantGender
/ORDER=ANALYSIS.

*Target Child Race

FREQUENCIES VARIABLES=ChildRace
/ORDER=ANALYSIS.
```

```
*Target Child Gender.

FREQUENCIES VARIABLES=ChildGender
/ORDER=ANALYSIS.

**********************************************
*Demographic Analysis by Complete / Withdraw
***********************************************

SORT CASES BY WITHDREW.
SPLIT FILE SEPARATE BY WITHDREW.

*Caregiver age.

DESCRIPTIVES VARIABLES=intake2
/STATISTICS=MEAN STDDEV MIN MAX

*Target Child Age

DESCRIPTIVES VARIABLES=intake6
/STATISTICS=MEAN STDDEV MIN MAX.

*Number of Children in Home Including Target Child.

DESCRIPTIVES VARIABLES=ChildrenInHome
/STATISTICS=MEAN STDDEV MIN MAX.

*Household Size Including Participant and Target Child.

DESCRIPTIVES VARIABLES=HouseholdToatal
/STATISTICS=MEAN STDDEV MIN MAX.

*Caregiver Race.

FREQUENCIES VARIABLES=ParticipantRace
/ORDER=ANALYSIS.

*Caregiver Gender.

FREQUENCIES VARIABLES=ParticipantGender
/ORDER=ANALYSIS.

*Target Child Race
```

```
FREQUENCIES VARIABLES=ChildRace
/ORDER=ANALYSIS.

*Target Child Gender.

FREQUENCIES VARIABLES=ChildGender
/ORDER=ANALYSIS.

SPLIT FILE OFF.

********************************************
*PRIMARY AND SECONDARY STUDY CONSTRUCTS
********************************************

******************************
*SERVICE BARRIERS CHECKLIST*
******************************

*Pre Total

RELIABILITY
/VARIABLES=sbc1_01 sbc2_01 sbc3_01 sbc4_01 sbc5_01 sbc6_01 sbc7_01 sbc8_01 sbc9_01
sbc10_01 sbc11_01 sbc12_01 sbc13_01 sbc14_01 sbc15_01 sbc16_01 sbc17_01 sbc18_01
sbc19_01 sbc20_01
sbc21_01 sbc22_01 sbc23_01
/SCALE('ALL VARIABLES') ALL
/MODEL=ALPHA.

*Post Total

RELIABILITY
/VARIABLES=sbc1_02 sbc2_02 sbc3_02 sbc4_02 sbc5_02 sbc6_02 sbc7_02 sbc8_02 sbc9_02
sbc10_02 sbc11_02 sbc12_02 sbc13_02 sbc14_02 sbc15_02 sbc16_02 sbc17_02 sbc18_02
sbc19_02 sbc20_02
sbc21_02 sbc22_02 sbc23_02
/SCALE('ALL VARIABLES') ALL
/MODEL=ALPHA.

*Pre Family

RELIABILITY
/VARIABLES=sbc1_01 sbc2_01 sbc3_01 sbc4_01 sbc5_01 sbc6_01 sbc7_01 sbc8_01 sbc9_01
/SCALE('ALL VARIABLES') ALL
/MODEL=ALPHA.
```

*Post Family

RELIABILITY
/VARIABLES=sbc1_02 sbc2_02 sbc3_02 sbc4_02 sbc5_02 sbc6_02 sbc7_02 sbc8_02 sbc9_02
/SCALE('ALL VARIABLES') ALL
/MODEL=ALPHA.

*Pre Provider

RELIABILITY
/VARIABLES=sbc16_01 sbc17_01 sbc18_01 sbc19_01 sbc20_01
/SCALE('ALL VARIABLES') ALL
/MODEL=ALPHA.
*Post Provider
RELIABILITY
/VARIABLES=sbc16_02 sbc17_02 sbc18_02 sbc19_02 sbc20_02
sbc21_02
/SCALE('ALL VARIABLES') ALL
/MODEL=ALPHA.

*Pre System

RELIABILITY
/VARIABLES=sbc22_01 sbc23_01
/SCALE('ALL VARIABLES') ALL
/MODEL=ALPHA.

*Post System

RELIABILITY
/VARIABLES=sbc22_02 sbc23_02
/SCALE('ALL VARIABLES') ALL
/MODEL=ALPHA.

*Pre Logistical

RELIABILITY
/VARIABLES=sbc10_01 sbc11_01 sbc12_01 sbc13_01 sbc14_01 sbc15_01
/SCALE('ALL VARIABLES') ALL
/MODEL=ALPHA.

*Post Logisitcal

```
RELIABILITY
/VARIABLES=sbc10_02 sbc11_02 sbc12_02 sbc13_02 sbc14_02 sbc15_02
/SCALE('ALL VARIABLES') ALL
/MODEL=ALPHA.

****************************
*PARENTING STRESS INDEX*
****************************

*Total PSI pre

RELIABILITY
/VARIABLES=psi1 psi2 psi3 psi4 psi5 psi6 psi7 psi8 psi9 psi10 psi11 psi12 psi13 psi14 psi15
psi16 psi17 psi18 psi19 psi20 psi21 psi22 psi23 psi24 psi25 psi26 psi27 psi28 psi29 psi30 psi31
psi32 psi33 psi34 psi35 psi36
/SCALE('ALL VARIABLES') ALL
/MODEL=ALPHA.

*Total PSI post

RELIABILITY
/VARIABLES=psi1_closing psi2_closing psi3_closing psi4_closing psi5_closing psi6_closing
psi7_closing psi8_closing psi9_closing psi10_closing psi11_closing psi12_closing psi13_closing
psi14_closing psi15_closing psi16_closing psi17_closing psi18_closing psi19_closing
psi20_closing psi21_closing psi22_closing psi23_closing psi24_closing psi25_closing
psi26_closing psi27_closing psi28_closing psi29_closing psi30_closing psi31_closing
psi32_closing psi33_closing psi34_closing psi35_closing psi36_closing
/SCALE('ALL VARIABLES') ALL
/MODEL=ALPHA.

*Parental Distress pre

RELIABILITY
/VARIABLES=psi1 psi2 psi3 psi4 psi5 psi6 psi7 psi8 psi9 psi10 psi11 psi12
/SCALE('ALL VARIABLES') ALL
/MODEL=ALPHA.

*Parental distress post

RELIABILITY
/VARIABLES=psi1_closing psi2_closing psi3_closing psi4_closing psi5_closing psi6_closing
psi7_closing psi8_closing psi9_closing psi10 /SCALE('ALL VARIABLES') ALL
/MODEL=ALPHA.
```

```
*Parent-Child Dysfunction Pre

RELIABILITY
/VARIABLES=psi13 psi14 psi15 psi16 psi17 psi18 psi19 psi20 psi21 psi22 psi23 psi24
/SCALE('ALL VARIABLES') ALL
/MODEL=ALPHA.

*Parent-Child Dysfunction Post

RELIABILITY
/VARIABLES=psi13_closing psi14_closing psi15_closing psi16_closing psi17_closing
psi18_closing psi19_closing psi20_closing psi21_closing psi22_closing psi23_closing
psi24_closing
/SCALE('ALL VARIABLES') ALL
/MODEL=ALPHA.

*Difficult Child Pre

RELIABILITY
/VARIABLES=psi25 psi26 psi27 psi28 psi29 psi30 psi31 psi32 psi33 psi34 psi35 psi36
/SCALE('ALL VARIABLES') ALL
/MODEL=ALPHA.

*Difficult Child Post

RELIABILITY
/VARIABLES=psi25_closing psi26_closing psi27_closing psi28_closing psi29_closing
psi30_closing psi31_closing psi32_closing psi33_closing psi34_closing psi35_closing
psi36_closing
/SCALE('ALL VARIABLES') ALL
/MODEL=ALPHA.

***********************************************
*STRENGTHS AND DIFFICULTIES QUESTIONNAIRE*
***********************************************

* Total Pre

RELIABILITY
/VARIABLES=sdq3 sdq8 sdq13 sdq16 sdq5 sdq7 sdq12 sdq18 sdq22 sdq2 sdq10 sdq15 sdq25
sdq6 sdq11 sdq14 sdq19 sdq23
/SCALE('ALL VARIABLES') ALL
/MODEL=ALPHA.
```

```
* Total Post

RELIABILITY
/VARIABLES=sdq3_v2 sdq8_v2 sdq13_v2 sdq16_v2 sdq5_v2 sdq7_v2 sdq12_v2 sdq18_v2
sdq22_v2 sdq2_v2 sdq10_v2 sdq15_v2 sdq25_v2 sdq6_v2 sdq11_v2 sdq14_v2 sdq19_v2
sdq23_v2
/SCALE('ALL VARIABLES') ALL
/MODEL=ALPHA.

*Externalizing Pre

RELIABILITY
/VARIABLES= sdq5 sdq7 sdq12 sdq18 sdq22 sdq22 sdq10 sdq15 sdq22
/SCALE('ALL VARIABLES') ALL
/MODEL=ALPHA.

*Externalizing Post

RELIABILITY
/VARIABLES= sdq5_v2 sdq7_v2 sdq12_v2 sdq18_v2 sdq22_v2 sdq22_v2 sdq10_v2 sdq15_v2
sdq22_v2
/SCALE('ALL VARIABLES') ALL
/MODEL=ALPHA.

*Internalizing Pre

RELIABILITY
/VARIABLES=sdq3 sdq8 sdq13 sdq16 sdq6 sdq11 sdq14 sdq19 sdq23
/SCALE('ALL VARIABLES') ALL
/MODEL=ALPHA.

*Internalizing Post

RELIABILITY
/VARIABLES=sdq3_v2 sdq8_v2 sdq13_v2 sdq16_v2 sdq6_v2 sdq11_v2 sdq14_v2 sdq19_v2
sdq23_v2
/SCALE('ALL VARIABLES') ALL
/MODEL=ALPHA.

****************
*SERVICE NEEDS*
****************

VARSTOCASES
```

```
  /MAKE GOALCOMPLETION FROM GOAL1 GOAL2 GOAL3
  /MAKE GOALTYPE FROM GOAL1TYPE GOAL2TYPE GOAL3TYPE
  /INDEX=Index1(3)
  /KEEP=pci intake2 intake3 ParticipantRace intake4 ParticipantGender intake6 intake7
ChildGender intake8 ChildRace ChildrenInHome OthersInHome HouseholdToatal
COMMUNITYTOTAL PRIMARYREASON ReligiousAffiliation TribalImmigrationHx psi1 psi2 psi3
psi4 psi5 psi6 psi7 psi8 psi9 psi10 psi11 psi12 psi13 psi14 psi15 psi16 psi17 psi18 psi19 psi20
psi21 psi22 psi23 psi24 psi25 psi26 psi27 psi28 psi29 psi30 psi31 psi32 psi33 psi34 psi35 psi36
psiident_closing psi1_closing psi2_closing psi3_closing psi4_closing
psi5_closing psi6_closing psi7_closing psi8_closing psi9_closing psi10_closing psi11_closing
psi12_closing psi13_closing psi14_closing psi15_closing psi16_closing psi17_closing
psi18_closing psi19_closing psi20_closing psi21_closing psi22_closing psi23_closing
psi24_closing psi25_closing psi26_closing psi27_closing psi28_closing psi29_closing
psi30_closing psi31_closing psi32_closing psi33_closing psi34_closing psi35_closing
psi36_closing defensive defensive1 parentaldistress parentaldistress1
parentchilddynsfunction parentchilddynsfunction1 difficultchild difficultchild1 totalpsi total1psi
drchange pdchange pcdchange dcchange totalchangepsi sdq1 sdq2 sdq3 sdq4 sdq5 sdq6 sdq7
sdq8 sdq9 sdq10 sdq11 sdq12 sdq13 sdq14 sdq15 sdq16 sdq17 sdq18 sdq19 sdq20 sdq21 sdq22
sdq23 sdq24 sdq25 sdq26 sdq27 sdq28 sdq29 sdq30 sdq31 sdq32 sdq33
initial_strengths_and_difficulties_questionnaire_complete sdqid_v2 sdq1_v2 sdq2_v2 sdq3_v2
sdq4_v2 sdq5_v2 sdq6_v2 sdq7_v2 sdq8_v2 sdq9_v2 sdq10_v2 sdq11_v2 sdq12_v2
sdq13_v2 sdq14_v2 sdq15_v2 sdq16_v2 sdq17_v2 sdq18_v2 sdq19_v2 sdq20_v2 sdq21_v2
sdq22_v2 sdq23_v2 sdq24_v2 sdq25_v2 sdq26_v2 sdq27_v2 sdq28_v2 sdq29_v2 sdq30_v2
sdq31_v2 sdq32_v2 sdq33_v2 emotion emotion1 conduct conduct1 hyper hyper1 peer peer1
prosocial prosocial1 totalsdq total1sdq externalizing externalizing1 internalizing internalizing1
totalchangesdq internalizingchange externalizingchange emotionchange conductchange
hyperchange peerchange prosocialchange sbc1_01 sbc2_01 sbc3_01 sbc4_01 sbc5_01
sbc6_01 sbc7_01 sbc8_01 sbc9_01 sbc10_01 sbc11_01 sbc12_01 sbc13_01 sbc14_01 sbc15_01
sbc16_01 sbc17_01 sbc18_01 sbc19_01 sbc20_01 sbc21_01 sbc22_01 sbc23_01 sbc1_02
sbc2_02 sbc3_02 sbc4_02 sbc5_02 sbc6_02 sbc7_02 sbc8_02 sbc9_02 sbc10_02 sbc11_02
sbc12_02 sbc13_02 sbc14_02 sbc15_02 sbc16_02 sbc17_02 sbc18_02 sbc19_02 sbc20_02
sbc21_02 sbc22_02 sbc23_02 TOTAL FAMILY_FACTORS LOGISTICAL_FACTORS
PROVIDER_FACTORS SYSTEM_FACTORS TOTAL1 FAMILY_FACTORS1 LOGISTICAL_FACTORS1
PROVIDER_FACTORS1 SYSTEM_FACTORS1
Navigator GOALPRE GOALTOTAL ADHERENCE MISSEDCHECKINS WITHDREW CHECKINS
  /NULL=KEEP.

***************************
*FREQUENCY OF SERVICE NEED*
***************************

FREQUENCIES VARIABLES=GOALTYPE
/ORDER=ANALYSIS.
```

```
******************************
*SERVICE ACCESS BY SERVICE NEED*
******************************

SELECT IF (WITHDREW=0).

CROSSTABS
  /TABLES=GOALCOMPLETION BY GOALTYPE
  /FORMAT=AVALUE TABLES
  /CELLS=COUNT
  /COUNT ROUND CELL.

SORT CASES BY pci Index1.
CASESTOVARS
  /ID=pci
  /INDEX=Index1
  /GROUPBY=VARIABLE.

*********************
*FSP GOAL COMPLETION*
*********************

FREQUENCIES VARIABLES=GOALTOTAL
/STATISTICS=MEAN STDDEV.

DESCRIPTIVES VARIABLES=GOALTOTAL
/STATISTICS=MEAN STDDEV.

FILTER OFF.
USE ALL.
EXECUTE.

*********************************************************
*PAIRED SAMPLES T-TESTS FOR PRIMARY AND SECONDARY OUTCOMES
*********************************************************

*************************************
*Family Service Plan Goal Completion
*************************************

T-TEST PAIRS=goaltotal WITH goalpre (PAIRED)
/ES DISPLAY(TRUE) STANDARDIZER(SD)
/CRITERIA=CI(.9500)
/MISSING=ANALYSIS.
```

```
**********************
*Service Barriers Checklist
**********************

T-TEST PAIRS=TOTAL1 FAMILY_FACTORS1 LOGISTICAL_FACTORS1 PROVIDER_FACTORS1
SYSTEM_FACTORS1 WITH TOTAL FAMILY_FACTORS LOGISTICAL_FACTORS PROVIDER_FACTORS
SYSTEM_FACTORS (PAIRED)
/ES DISPLAY(TRUE) STANDARDIZER(SD)
/CRITERIA=CI(.9500)
/MISSING=ANALYSIS.

**********************
*Parenting Stress Index
**********************

T-TEST PAIRS= parentaldistress1 parentchilddynsfunction1 difficultchild1 total1psi
WITH parentaldistress parentchilddynsfunction difficultchild totalpsi
(PAIRED)
/ES DISPLAY(TRUE) STANDARDIZER(SD)
/CRITERIA=CI(.9500)
/MISSING=ANALYSIS.

************************************
*Strengths and Difficulties Questionnaire
************************************

T-TEST PAIRS=total1sdq
internalizing1 externalizing1 hyper1 conduct1 peer1 prosocial1 emotion1 WITH totalsdq
internalizing externalizing hyper conduct peer prosocial emotion (PAIRED)
/ES DISPLAY(TRUE) STANDARDIZER(SD)
/CRITERIA=CI(.9500)
/MISSING=ANALYSIS.

************************************************************
*ATTRITION, NUMBER OF CHECK INS, NAVIGATOR ADHERENCE*
************************************************************

*Attrition

FREQUENCIES VARIABLES=WITHDREW
/STATISTICS=MEAN STDDEV.
```

*Intervention engagement

FREQUENCIES VARIABLES=MISSEDCHECKINS
/STATISTICS=MEAN STDDEV.
DESCRIPTIVES VARIABLES=MISSEDCHECKINS
/STATISTICS=MEAN STDDEV MIN MAX.
FREQUENCIES VARIABLES=CHECKINS
/STATISTICS=MEAN STDDEV.
DESCRIPTIVES VARIABLES=CHECKINS
/STATISTICS=MEAN STDDEV MIN MAX

*Navigator Protocol Adherence

DESCRIPTIVES VARIABLES=ADHERENCE
/STATISTICS=MEAN STDDEV MIN MAX

****
RECORD OF CONSTRUCT DEVELOPMENT FOR EACH SCALE AND SUBSCALE
****

****
*SERVICE BARRIERS CHECKLIST
****
*Recode "not present" variable from 0 = present, 1 = not present to, 0 = not present, and 1 = present
RECODE sbc1_01 sbc2_01 sbc3_01 sbc4_01 sbc5_01 sbc6_01 sbc7_01 sbc8_01 sbc9_01 sbc10_01 sbc11_01 sbc12_01 sbc13_01 sbc14_01 sbc15_01 sbc16_01 sbc17_01 sbc18_01 sbc19_01 sbc20_01
sbc21_01 sbc22_01 sbc23_01 (0=1) (1=0).
EXECUTE.
RECODE sbc1_02 sbc2_02 sbc3_02 sbc4_02 sbc5_02 sbc6_02 sbc7_02 sbc8_02 sbc9_02 sbc10_02 sbc11_02 sbc12_02 sbc13_02 sbc14_02 sbc15_02 sbc16_02 sbc17_02 sbc18_02 sbc19_02 sbc20_02
sbc21_02 sbc22_02 sbc23_02 (0=1) (1=0).
EXECUTE.
*PRE-TEST
COMPUTE TOTAL=sbc1_01 + sbc2_01 + sbc3_01 + sbc4_01 + sbc5_01 + sbc6_01 + sbc7_01 + sbc8_01 + sbc9_01 +
sbc10_01 + sbc11_01 + sbc12_01 + sbc13_01 + sbc14_01 + sbc15_01 + sbc16_01 + sbc17_01 + sbc18_01 + sbc19_01 + sbc20_01 +
sbc21_01 + sbc22_01 + sbc23_01.
EXECUTE.
COMPUTE FAMILY_FACTORS=sbc1_01 + sbc2_01 + sbc3_01 + sbc4_01 + sbc5_01 + sbc6_01 + sbc7_01 + sbc8_01 + sbc9_01.

```
EXECUTE.
COMPUTE LOGISTICAL_FACTORS=sbc10_01 + sbc11_01 + sbc12_01 + sbc13_01 + sbc14_01 +
sbc15_01.
EXECUTE.
COMPUTE PROVIDER_FACTORS=sbc16_01 + sbc17_01 + sbc18_01 + sbc19_01 + sbc20_01 +
sbc21_01.
EXECUTE.
COMPUTE SYSTEM_FACTORS=sbc22_01 + sbc23_01.
EXECUTE.
*POST TEST
COMPUTE TOTAL1=sbc1_02 + sbc2_02 + sbc3_02 + sbc4_02 + sbc5_02 + sbc6_02 + sbc7_02 +
sbc8_02 + sbc9_02 +
sbc10_02 + sbc11_02 + sbc12_02 + sbc13_02 + sbc14_02 + sbc15_02 + sbc16_02 + sbc17_02
+ sbc18_02 + sbc19_02 + sbc20_02 +
sbc21_02 + sbc22_02 + sbc23_02.
EXECUTE.
COMPUTE FAMILY_FACTORS1=sbc1_02 + sbc2_02 + sbc3_02 + sbc4_02 + sbc5_02 + sbc6_02 +
sbc7_02 + sbc8_02 + sbc9_02.
EXECUTE.
COMPUTE LOGISTICAL_FACTORS1=sbc10_02 + sbc11_02 + sbc12_02 + sbc13_02 + sbc14_02 +
sbc15_02.
EXECUTE.
COMPUTE PROVIDER_FACTORS1=sbc16_02 + sbc17_02 + sbc18_02 + sbc19_02 + sbc20_02 +
sbc21_02.
EXECUTE.
COMPUTE SYSTEM_FACTORS1=sbc22_02 + sbc23_02.
EXECUTE.
**********************************************
*STRENGTHS AND DIFFICULTIES QUESTIONNAIRE*
**********************************************
COMPUTE emotion=sdq3 + sdq8 + sdq13 + sdq16.
EXECUTE.
COMPUTE emotion1=sdq3_v2 + sdq8_v2 + sdq13_v2 + sdq16_v2.
EXECUTE.
COMPUTE conduct=sdq5 + sdq7 + sdq12 + sdq18 + sdq22.
EXECUTE.
COMPUTE conduct1=sdq5_v2 + sdq7_v2 + sdq12_v2 + sdq18_v2 + sdq22_v2.
EXECUTE.
COMPUTE hyper=sdq2 + sdq10 + sdq15 + sdq21 + sdq25.
EXECUTE.
COMPUTE hyper1=sdq2_v2 + sdq10_v2 + sdq15_v2 + sdq21_v2 + sdq25_v2.
EXECUTE.
COMPUTE peer=sdq6 + sdq11 + sdq14 + sdq19 + sdq23.
EXECUTE.
```

```
COMPUTE peer1=sdq6_v2 + sdq11_v2 + sdq14_v2 + sdq19_v2 + sdq23_v2.
EXECUTE.
COMPUTE prosocial=sdq1 + sdq4 + sdq9 + sdq17 + sdq20.
EXECUTE.
COMPUTE prosocial1=sdq1_v2 + sdq4_v2 + sdq9_v2 + sdq17_v2 + sdq20_v2.
EXECUTE.
COMPUTE total=sdq3 + sdq8 + sdq13 + sdq16 + sdq5 + sdq7 + sdq12 + sdq18 + sdq22 + sdq2 +
sdq10 + sdq15 + sdq21 + sdq25 + sdq6 + sdq11 + sdq14 + sdq19 + sdq23.
EXECUTE.
COMPUTE total1=sdq3_v2 + sdq8_v2 + sdq13_v2 + sdq16_v2 + sdq5_v2 + sdq7_v2 + sdq12_v2
+ sdq18_v2 + sdq22_v2 + sdq2_v2 + sdq10_v2 + sdq15_v2 + sdq21_v2 + sdq25_v2 + sdq6_v2 +
sdq11_v2 + sdq14_v2 + sdq19_v2 + sdq23_v2.
EXECUTE.
COMPUTE externalizing=conduct + hyper.
EXECUTE.
COMPUTE externalizing1=conduct1 + hyper1.
EXECUTE.
COMPUTE internalizing=emotion + peer.
EXECUTE.
COMPUTE internalizing1=emotion1 + peer1.
EXECUTE.
*CHANGE SCORE
COMPUTE totalchange=total1 - total.
EXECUTE.
COMPUTE internalizingchange=internalizing1 - internalizing.
EXECUTE.
COMPUTE externalizingchange=externalizing1 - externalizing.
EXECUTE.
COMPUTE emotionchange=emotion1 - emotion.
EXECUTE.
COMPUTE conductchange=conduct1 - conduct.
EXECUTE.
COMPUTE hyperchange=hyper1 - hyper.
EXECUTE.
COMPUTE peerchange=peer1 - peer.
EXECUTE.
COMPUTE prosocialchange=prosocial1 - prosocial.
EXECUTE.
***************************
*PARENTING STRESS INDEX*
****************************
COMPUTE defensive=psi1 + psi2 + psi3 + psi7 + psi8 + psi9 + psi11.
EXECUTE.
COMPUTE defensive1=psi1_closing + psi2_closing + psi3_closing + psi7_closing + psi8_closing +
```

```
psi9_closing + psi11_closing.
EXECUTE.
COMPUTE parentaldistress=psi1 + psi2 + psi3 + psi4 + psi5 + psi6 + psi7 + psi8 +psi9 +psi10
+psi11 + psi12.
EXECUTE.
COMPUTE parentaldistress1=psi1_closing + psi2_closing + psi3_closing + psi4_closing +
psi5_closing + psi6_closing + psi7_closing + psi8_closing +psi9_closing +psi10_closing
+psi11_closing + psi12_closing.
EXECUTE.
COMPUTE parentchilddysfunction=psi13 + psi14 + psi15 + psi16 + psi17 + psi18 + psi19 + psi20
+psi21 +psi22 +psi23 + psi24.
EXECUTE.
COMPUTE parentchilddysfunction1=psi13_closing + psi14_closing + psi15_closing +
psi16_closing + psi17_closing + psi18_closing + psi19_closing + psi20_closing +psi21_closing
+psi22_closing +psi23_closing + psi24_closing.
EXECUTE.
COMPUTE difficultchild=psi25+ psi26 + psi27 + psi28 + psi29 + psi30 + psi31 + psi32 +psi33
+psi34 +psi35 + psi36.
EXECUTE.
COMPUTE difficultchild1=psi25_closing + psi26_closing + psi27_closing + psi28_closing +
psi29_closing + psi30_closing + psi31_closing + psi32_closing +psi33_closing +psi34_closing
+psi35_closing + psi36_closing.
EXECUTE.
COMPUTE total=parentaldistress + parentchilddysfunction + difficultchild.
EXECUTE.
COMPUTE total1=parentaldistress1 + parentchilddysfunction1 + difficultchild1.
EXECUTE.
*CHANGE SCORE
COMPUTE drchange=defensive1 - defensive.
EXECUTE.
COMPUTE pdchange=parentaldistress1 - parentaldistress.
EXECUTE.
COMPUTE pcdchange=parentchilddysfunction1 - parentchilddysfunction.
EXECUTE.
COMPUTE dcchange=difficultchild1 - difficultchild.
EXECUTE.
COMPUTE totalchange=total1 - total.
EXECUTE.
```
