# Supplementary material for: Promoting health and social equity through family navigation to prevention and early intervention services: a proof of concept study
Source: BMC Public Health. 2022 Oct 27;22:1972. doi: 10.1186/s12889-022-14320-4 (PMC9610316; doi:10.1186/s12889-022-14320-4)
Supplement: Supplementary file 7 — Supplementary Material 7 [file 12889_2022_14320_MOESM7_ESM.pdf]

```
*Promoting health and social equity through family navigation to prevention and early
intervention services: A proof of concept study
 *Principal Investigator: Jeffrey Waid
 *Contact email: jdwaid@umn.edu

*********************
*NAVIGATOR EFFORT
*********************

*Project Activitiy

FREQUENCIES VARIABLES=Category
  /ORDER=ANALYSIS.

*Time Devoted to Each Project Activity

MEANS TABLES=Timemin BY Category
  /CELLS=MEAN STDDEV MIN MAX
```
